# In Vitro and In Vivo Antioxidant Activities of the Flowers and Leaves from *Paeonia rockii* and Identification of Their Antioxidant Constituents by UHPLC-ESI-HRMS^n^ via Pre-Column DPPH Reaction

**DOI:** 10.3390/molecules23020392

**Published:** 2018-02-09

**Authors:** Yating Bao, Yan Qu, Jinhua Li, Yanfang Li, Xiaodong Ren, Katherine G. Maffucci, Ruiping Li, Zhanguo Wang, Rui Zeng

**Affiliations:** 1 College of Pharmacy, Southwest Minzu University, Chengdu 610041, China; 13678027728@163.com (Y.B.); 15680690159@163.com (J.L.); yanfangli8225@126.com (Y.L.); 2 Pharmacy College, Chengdu University of Traditional Chinese Medicine, Chengdu 611137, China; 3 Department of Chemistry, University of California, Riverside, CA 92521, USA; xiadong@ucr.edu; 4 Department of Chemistry, Stony Brook University, Stony Brook, NY 11794, USA; katherine.maffucci@stonybrook.edu; 5 Metabonomics Synergy Innovation Laboratory, School of Medicine and Nursing, Chengdu University, Chengdu 610106, China; liruiping11@163.com (R.L.); wangzhanguo@scu.edu.cn (Z.W.)

**Keywords:** *Paeonia rockii*, antioxidant activity, total phenolic content, chemical composition, UHPLC-ESI-HRMS^n^

## Abstract

The genus *Paeonia*, also known as the “King of Flowers” in China, is an important source of traditional Chinese medicine (TCM). Plants of this genus have been used to treat a range of cardiovascular and gynecological diseases. However, the potential pharmacological activity of one particular species, *Paeonia rockii*, has not been fully investigated. In the first part of the present study, 2,2-diphenyl-1-picrylhydrazyl (DPPH), 2,2′-azino-bis-(3-ethylbenzothiazoline-6-sulfonic) acid (ABTS), reducing power assays, and metal ion chelating assays were used to investigate the in vitro antioxidant activities of *Paeonia rockii*. In the second portion of the study, a mouse model of d-galactose-induced aging was used to validate the antioxidant effects of the flowers from *Paeonia rockii* in vivo. Lastly, potential antioxidant constituents were screened and identified by ultra-high pressure liquid chromatography and electrospray ionization coupled with high-resolution mass spectrometry (UHPLC-ESI-HRMS^n^) combined with the DPPH assay. Results indicated that the flowers and leaves exhibited stronger antioxidant activity than ascorbic acid in vitro. The therapeutic effect of *Paeonia* *rockii* was determined in relation to the levels of biochemical indicators, such as 8-iso-prostaglandin F_2α_ (8-iso PGF_2α_) in the serum, superoxide dismutase (SOD), protein carbonyl, malondialdehyde (MDA), and glutathione (GSH) in the liver and brain, after daily intra-gastric administration of different concentrations of extracts (100, 200 and 400 mg/kg) for three weeks. The levels of 8-iso PGF_2α_ (*p* < 0.01) and protein carbonyl groups (*p* < 0.01) were significantly reduced, whereas those of SOD (*p* < 0.05) had significantly increased, indicating that components of the flowers of *Paeonia rockii* had favorable antioxidant activities in vivo. Furthermore, UHPLC-ESI-HRMS^n^, combined with pre-column DPPH reaction, detected 25 potential antioxidant compounds. Of these, 18 compounds were tentatively identified, including 11 flavonoids, four phenolic acids, two tannins, and one monoterpene glycoside. This study concluded that the leaves and flowers from *Paeonia rockii* possess excellent antioxidant properties, highlighting their candidacy as “new” antioxidants, which can be utilized therapeutically to protect the body from diseases caused by oxidative stress.

## 1. Introduction

Oxidative stress is an excess of free radical production caused by a series of complex cellular processes, such as the accumulation of hydrogen peroxide, hydroxyl radicals, and superoxide anions during normal cellular metabolism. However, oxidative stress is thought to be involved in the development of a range of human diseases including aging, liver damage, cardiovascular disease, and cancer [1,2,3,4]. According to previous reports, antioxidants are able to prevent the production of free radicals [5,6] and the chain reaction which occurs before vital molecules are damaged, thus protecting the body from subsequent oxidative stress and further tissue injury [7,8]. There are two basic categories of antioxidants: synthetic and natural. In general, synthetic antioxidants are compounds with phenolic structures of varying degrees of alkyl substitution, while natural antioxidants may be flavonoids and phenolic compounds [9]. Due to the toxicity of synthetic antioxidants and their undesirable effects on human health [10], interest in natural antioxidants has increased considerably [11]. Natural antioxidants originating from Traditional Chinese medicinal herbs *Bletilla striata*, *Lonicera japonica*, and *Paeonia* have been reported to possess favorable antioxidant activity [12,13,14].

The genus *Paeonia* features 35 species [15], originates from Europe and Asia and is referred to as the “King of Flowers” in China. *Paeonia* is an ornamental shrub and one of the most important sources of traditional Chinese medicine (TCM) [14]. *Paeonia* predominantly contain monoterpenes, triterpenoids, flavonoids, phenols, and tannins [16,17] and exhibit antioxidative, anti-inflammatory, antimicrobial, analgesic, sedative, cardioprotective, and gynecological effects [18,19]. Earlier studies have isolated and identified five flavonoids from the flowers of *Paeonia ostii*, which are responsible for its strong antioxidant activities [20]. Seeds from the nine tree peony species have been shown to contain phenolic compounds which confer antioxidant activity, and have subsequently gained attention as a health food [21]. The chemical components and pharmacological activities of *Paeonia* roots have also been investigated recently, including its antioxidant activity [17,22].

*Paeoni rockii* (S.G. Haw & Lauener) T. Hong & J.J. Li ex D.Y. Hong (*Paeoniaceae*) is an important ancestral species of cultivated tree peony which naturally thrives in the provinces of Gansu, Henan, and Sichuan, Tibet, and several regions in Northern China. *Paeonia rockii* grows in areas at an altitude of 1100–2800 m [23]. Although local indigenous people use the flowers and leaves from *Paeonia rockii* as a decoction for the treatment of acne and gynecological diseases, its application in other medical scenarios has yet to be documented. Existing studies on the efficacy of *Paeonia rockii* suggest that its roots and seeds contain antioxidant compounds; oil extract from seeds, fruits, and root bark is already utilized as a component of moisturizing cosmetics and health-care capsules and as a new health food [14,24,25]. Given the rarity of *Paeonia rockii*, it is important to carry out specific studies of its flowers and leaves in order to make better use of this important resource.

However, information relating to the antioxidant activities of the flowers and leaves from *Paeonia rockii* is very scarce. Therefore, this study aims to investigate the potential antioxidant activities of leaves and flowers from *Paeonia rockii*, and to identify antioxidant compounds by UHPLC-ESI-HRMS^n^ with a pre-column DPPH reaction.

## 2. Results

### 2.1. Total Phenolic and Flavonoid Content

Most antioxidant activities from plant sources correlate with phenolic and flavonoid contents [26,27,28]. In this study, we determined the TPC and TFC of MF and ML (Table 1). The TPC of ML at 693.93 ± 0.82 mg GAE/g extract was higher than that of MF at 540.38 ± 1.83 mg GAE/g, whereas the TFC of MF at 130.40 ± 0.41 mg RE/g extract was lower than that of ML at 180.8 ± 0.37 mg RE/g extract. TPC was also higher than TFC in both MF and ML extracts.

### 2.2. In Vitro Assays

#### 2.2.1. DPPH Radical Scavenging Activity

**Figure 1:**
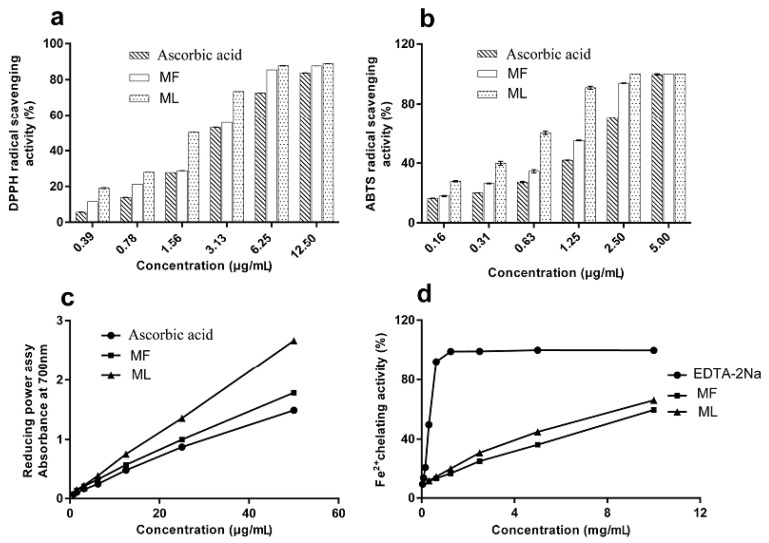
Determination of the antioxidant activities of extracts from *Paeonia rockii* leaves and flowers using (**a**) DPPH radical scavenging assay; (**b**) ABTS radical scavenging assay; (**c**) reducing power assay; and (**d**) Fe^2+^ chelating assay.

The DPPH radical is a stable free radical which is widely used in the evaluation of antioxidant capacity [29]. In the present study, the DPPH radical-scavenging activity of extracts was measured using spectrometric methods. Figure 1a shows dose-dependent activity, while Table 1 shows the IC_50_; a low IC_50_ value indicates high antioxidant activity. The DPPH radical-scavenging activity can be represented as follows: ML > MF > ascorbic acid.

#### 2.2.2. ABTS Radical Scavenging Activity

The ABTS radical cation is generated when the ABTS radical cation reacts with oxidants and its absorbance is measure at 734 nm [5]. Antioxidants from *Paeonia rockii* extracts are known to reduce the ABTS radical cation. Figure 1b presents the ABTS radical-scavenging activity of *Paeonia rockii* extracts while Table 1 shows the relevant IC_50_ values. The ABTS radical-scavenging activity is as follows: ML > MF > ascorbic acid. Hence, *Paeonia rockii* extracts exhibit excellent ABTS radical cation scavenging ability.

#### 2.2.3. Reducing Power

The reducing power assay determines antioxidant activity based on the reducing potential, or the ability of the extract to convert Fe^3+^ to Fe^2+^ [30]. The reducing potential was monitored by measuring the formation of a Prussian blue product at 700 nm. Figure 1c shows that the reducing power of MF and ML extracts increased with increasing concentrations. The ascorbic acid equivalents (AAE) given in μg AAE/100 μg of MF and ML were 119.85 ± 0.00 and 178.81 ± 0.00, respectively. The order of reducing power was ML > MF > ascorbic acid, indicating that ML had a higher antioxidant activity than MF and ascorbic acid.

#### 2.2.4. Metal Ion Chelating Ability

Metal chelation is an important antioxidant property, as it reduces the concentration of the catalyzing transition metal in lipid peroxidation [31,32]. In this study, EDTA-Na_2_ was used as a positive control. ML showed a significantly stronger Fe^2+^ chelating activity than MF (*p* < 0.05). Figure 1d shows the Fe^2+^ chelating activity as EDTA-Na_2_ > ML > MF. The values demonstrate that EDTA has the strongest chelating capacity and the chelation rate than ML and MF. The results suggest that the ML and MF complexes with the ferrous ion in a dose-dependent manner [32].

### 2.3. In Vivo Assays

#### 2.3.1. Establishment of an Animal Model

As shown in Table 2 and Table 3, the levels of 8-iso PGF2α in serum, and the levels of MDA and protein carbonyl in the liver and brain of the d-galactose-induced group of mice were significantly increased (*p* < 0.01), except for MDA in the liver (*p* < 0.05). MDA and protein carbonyl were reported as the major marker of endogenous lipid peroxidation, the increase of the levels of MDA and protein carbonyl showed d-galactose-induced benefit to the degree of oxidation [33,34]. Furthermore, SOD and GSH levels in the liver and brain of the d-galactose group were significantly reduced (*p* < 0.01), except for GSH in the brain (*p* < 0.05). This indicates that d-galactose can cause oxidative stress or damage.

#### 2.3.2. Biochemical Indices

The in vivo antioxidant effects of treatment with extracts prepared from the flowers of *Paeonia rockii* are shown in Table 2. Notably, treatment significantly reversed the antioxidant activity of 8-iso PGF2α, MDA and protein carbonyl in d-galactose-induced mice. Of these indices, 8-iso PGF2α in the serum, and protein carbonyl in the liver and brain were the biochemical markers indicative of the most extensive change. Moreover, treatment with flower extract markedly increased the levels of antioxidants, including SOD and GSH, compared with the d-galactose-induced model mice, especially in terms of SOD in the liver and brain (Table 3). The flower extract reduced oxidative damage by changing the levels of important biochemical indices, presumably due to its powerful antioxidant properties. The phytochemicals found to be present in the flower extracts, such as flavonoids and phenolic components, are likely to be responsible for the observed antioxidant properties, which have been reported previously.

### 2.4. Screening Antioxidants by DPPH-UHPLC-ESI-HRMS^n^ Analysis

**Figure 2:**
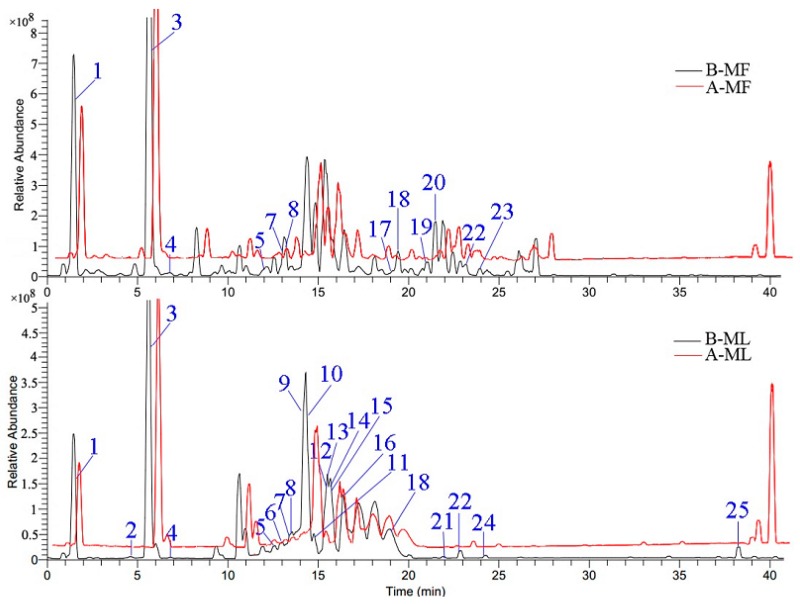
Base peak chromatogram (BPC) of the extracts from *Paeonia rockii* flowers and leaves (negative mode). B-MF: Methanol extracts from flowers without reaction with DPPH; A-MF: Methanol extracts from flowers reacted with DPPH; B-ML: Methanol extracts from leaves without reaction with DPPH; A-ML: Methanol extracts of leaves after reaction with DPPH.

DPPH-UHPLC-ESI-HRMS^n^ can be used for the rapid screening of antioxidants from complex mixtures, based on the fact that the level of antioxidant compounds decreases or disappears completely following the pre-column reaction with DPPH [35,36]. In the current study, potential antioxidant compounds were identified by UHPLC-ESI-HRMS^n^ in negative ion mode, and their retention time (t_R_), molecular formula, and MS fragmentation patterns were compared with published data, MS/MS data and identified compounds were presented in Table 4. Figure 2 shows chromatograms generated by extracts prepared from the flowers and leaves from *Paeonia rockii* before and after reaction with a DPPH radical. Twenty-five potential antioxidant compounds were screened based on a 46 compound database created in a preliminary study carried out by our group [37]; 18 of these compounds were identified in *Paeonia rockii*. Of these, 11 (quercetin-7-*O*-glucoside, quercetin-3-*O*-glucoside, kaempferol-7-*O*-glucoside, kaempferol galloylglucoside, astragalin, apigenin rhamnoglucoside, isorhamnetin-3-*O*-glucoside, apigenin galloylglucoside isomer, isorhamnetin, and apigenin), are flavonoids; four (gallic acid, hydroxybenzoic acid, methyl gallate, and methyl digallate) are phenolic acids; two (digalloyl glucose and tetragalloylglucose) are tannins; and one, benzoyloxypaeoniflorin, is the only monoterpene glycoside [17,37]. The results arising from the in vitro antioxidant activity tests, and the DPPH-UHPLC-ESI-HRMS^n^ assay, show that leaves have stronger antioxidant activity than flowers—this may be owing to the structure and concentration of the individual constituents.

## 3. Discussion

Previous reports showed that extracts prepared from *Paeonia* seeds and roots exhibited antioxidant activity [21,22,38] and that seeds can be used as a source of functional food [25,39]. However, the biological activities of the *Paeonia rockii* plant have been scarcely investigated, and this study is the first to establish that *Paeonia rockii* leaf and flower extracts possess excellent DPPH radical scavenging activity, ABTS radical scavenging activity, and reducing power. In comparison with other members of the *Paeonia* genus, the antioxidant activity of flowers from *Paeonia rockii* was superior to that of *Paeonia ostii* and other *Paeonia* [20,40,41]. Earlier reports demonstrated the IC_50_ values of *Paeonia ostii* in DPPH and ABTS radical scavenging assays were in the range of 20 µg/mL and 40 µg/mL, respectively [21]. In this study, the IC_50_ values of *Paeonia rockii* in DPPH and ABTS radical scavenging assay were lower (3 µg/mL). Compared with *Paeonia rockii* roots, the extracts of flowers and leaves also demonstrated a stronger DPPH radical scavenging effect (13.3 µg/mL) [14]. Moreover, the antioxidant ability of extracts from flowers and leaves in vitro was stronger than that of ascorbic acid.

In the present study, the flowers and leaves from *Paeonia rockii* contain large amounts of total flavonoids and total phenolic acids. Flavonoids and phenolic acids are known to act as antioxidants, not only for their ability to donate hydrogen or electrons, but also for their role as stable radical intermediates [42]. Other previous results revealed that the radical scavenging activities of the flavonoids tested were correlated with the number and position of phenolic hydroxyl groups in the molecules [42]. Furthermore, the presence of at least one ortho-dihydroxy group in the benzene ring of the polyphenols was involved in the stability of electrons and free radicals [43]. DPPH-UHPLC-ESI-HRMS^n^ assay confirmed that flavonoids and phenolic acids play a certain role in the extract’s antioxidant activity.

DPPH is one of the known stable radical species used to measure the radical-scavenging potential of various antioxidants, changing the molecular structure of the antioxidant in the process [44,45]. The DPPH-HPLC method can be used as a rapid screening tool for radical scavenging in complex mixtures, particularly plant extracts with minimal sample preparation. It was hypothesized that the antioxidant structure would change following a reaction with DPPH. Thus, the peak areas of compounds would be reduced or disappear completely [35,36,46]. As seen from the DPPH-UHPLC-ESI-HRMS^n^ assay, our data highlights potential antioxidant compounds containing 11 flavonoids, four phenolic acids, two tannins, and one monoterpene glycoside. It is likely that flavonoids and phenolic acids represent the major compounds responsible for the antioxidant activity of *Paeonia rockii* flowers and leaf extracts [14,47].

Previous studies demonstrated that 10 of the 18 potential antioxidant compounds identified using the DPPH-UHPLC-ESI-HRMSn assay showed significant antioxidant activity by in vitro assessments: quercetin [21], gallic acid, methyl gallate [14], apigenin [36], methyl digallate [48], isorhamnetin [49], kaempferol-7-*O*-glucoside, isorhamnetin-3-*O*-glucoside [50], quercetin-7-*O*-glucoside, and quercetin-3-*O*-glucoside [51]. These results are consistent with previous reports. Notably, gallic acid, the ortho-hydroxyl group on the benzene ring, was shown to be the active group and the more hydroxyl groups, the stronger the oxidation resistance [43]. Furthermore, eight potential antioxidant components were identified in *Paeonia rockii* for the first time in this study: hydroxybenzoic acid, astragalin, benzoyloxypaeoniflorin, tetragalloylglucose, apigenin galloylglucoside isomer, apigenin rhamnoglucoside, kaempferol galloylglucoside, isorhamnetin-3-*O*-glucoside, and digalloyl glucose. UHPLC-ESI-HRMS^n^ with a pre-column DPPH reaction as a method to identify antioxidant compounds from complex natural extraction is fast, low cost, high-throughput, and yields an index value [36]. In addition, qualitative and quantitative analyses of the antioxidant components, along with intensive studies of the mechanisms involved, are necessary.

In this study, in vivo antioxidant activity was validated using an established mouse model of d-galactose-induced aging to determine the levels of serum 8-iso PGF2α, SOD, protein carbonyl, MDA, and levels of GSH in the liver and brain as biochemical indicators. The d-galactose-induced aging mouse model has been utilized extensively for evaluating the antioxidant effects of drugs and plant extracts [33,34,52]. d-galactose treatment can increase the levels of 8-iso PGF2α, MDA, and protein carbonyl, and reduce the levels of SOD and GSH [34,53]. Antioxidants can prevent increases in the levels of biochemical indicators resulting from d-galactose treatment [33]. Extracts from *Paeonia rockii* flowers decreased levels of the biochemical indicators which had increased following d-galactose induction, particularly levels of 8-iso PGF2α, protein carbonyl, and SOD. In the present study, extracts of flowers from *Paeonia rockii* showed antioxidant activity in vivo. However, whether the 18 potential antioxidant compounds in vitro are effective antioxidants in vivo remains to be seen. Furthermore, studies are needed to isolate and identify the significant activities of compounds present in extracts of *Paeonia rockii* flowers and leaves and to elucidate their molecular mechanisms. Several previous in-depth investigations have indicated that oxidative stress was related to inflammation, cardiovascular disease, and Alzheimer’s disease [54,55,56]; moreover, biochemical indicators, such as increased GSH and SOD, and reduced levels of MDA, were identified as effective indices of inflammation and cardiovascular disease. Therefore, effective antioxidant activities in natural products and resources could play an important role in the prevention or treatment of diseases caused by oxidative stress.

## 4. Material and Methods

### 4.1. Chemicals and Reagents

The following chemicals were purchased from Chengdu Must Biotechnology Co., Ltd. (Chengdu, China): quercetin-3-*O*-glucoside, kaempferol, isorhamnetin, DPPH, ABTS, Folin-Ciocalteu reagent, and ascorbic acid. All other analytical reagents used for plant extraction and antioxidant analyses were obtained from Chengdu Kelong Chemical Co., Ltd. (Chengdu, China). UHPLC-ESI-HRMS^n^ analysis was carried out with HPLC-grade acetonitrile (Sigma-Aldrich, St. Louis, MO, USA) and deionized water purified using a Milli-Q system (Millipore, Bedford, MA, USA).

### 4.2. Plant Material and Extraction

Samples of *Paeonia rockii* were collected from Songpan County (Northwestern Sichuan, China, and Tibetan areas) in May 2014, and the plants were identified by Professor Linfang Huang from the Institute of Medicinal Plant Development, Peking Union Medical College and Chinese Academy of Medical Sciences (Beijing 100193, China). The plant material consisted of individually-separated leaves and flowers and was preserved in the Herbarium of Southwest Minzu University.

The leaves and flowers were initially freeze-dried and then ground to a fine powder in a mechanical grinder with a 65-µm mesh size. To prepare extracts, 100 g of the plant powder was sonicated three times at room temperature for 1 h with 1000 mL of methanol. Filtered solutions were then concentrated using a rotary evaporator. The residue was lyophilized, and the resulting dry powder was stored at 4 °C. Methanol extracts of *Paeonia rockii* flowers (MF) and leaves (ML) yielded 20.53% and 19.62% dry matter, respectively, relative to the dry starting material. For UHPLC-ESI-HRMS^n^ analysis, and in vitro studies, the dry extracts were re-dissolved in methanol (to a concentration of 5 mg/mL) and filtered through 0.22-μm nylon microporous membranes.

### 4.3. Determination of Total Phenolic Content (TPC)

The total phenolic content (TPC) of each extract was determined using Folin-Ciocalteu reagent according to a previously described method [5,57] with minor modifications. First, 0.2 mL of each sample (0.1 mg/mL) or standard (gallic acid; 3.125–100 μg/mL) was mixed with 2 mL of Folin-Ciocalteu reagent. After 5 min, 4 mL of 10% Na_2_CO_3_ was then added to the mixture, which was incubated at room temperature for 120 min before the absorbance was measured at 765 nm. TPC was expressed as mg gallic acid equivalents (GAE)/g dry weight of the extracts.

### 4.4. Determination of Total Flavonoid Content (TFC)

The total flavonoid content (TFC) of MF and ML was determined by a colorimetric assay using rutin as a standard [58]. Briefly, 5 mL of MF and ML (0.1 mg/mL) or rutin (8–48 μg/mL) was mixed with 1 mL of 5% NaNO_2_. After 6 min, 1 mL of 10% aluminum nitrate was added, and the mixture was allowed to stand for another 6 min. Afterwards, 10 mL of 10% NaOH was added to the mixture. The absorbance was measured at 510 nm after incubating the mixture at room temperature for 15 min. TFC was expressed as mg rutin equivalents (RE)/g dry weight of the extracts.

### 4.5. In Vitro Antioxidant Activity Testing

#### 4.5.1. DPPH Radical Scavenging Assay

The DPPH radical-scavenging activity assay was performed as previously reported [29]. The extracts (2 mL) were mixed at different concentrations (0.39–12.50 μg/mL), with 2 mL of DPPH solution (0.1 mM, in methanol). The mixture was then shaken and incubated at room temperature for 30 min, and the absorbance was measured at 517 nm. Ascorbic acid was used as a reference [19,28]. Then, radical scavenging activity was calculated as follows:

DPPH radical scavenging activity (%) = [1 − (*Ai* − *As*)/*Ac*] × 100

where *Ai* is the absorbance in the presence of the extract. *As* is the absorbance in the presence of the sample background; and *Ac* is the absorbance of the negative control (without the extracts). IC_50_ was determined by probit regression using IBM’s Statistical Program for Social Sciences (SPSS, IBM, Armonk, NY, USA) analysis of variance (20.0).

#### 4.5.2. ABTS Radical Scavenging Assay

The ability of the extracts to scavenge ABTS radical cations was evaluated using a previously described method [5] with some modifications. First, an ABTS radical cation solution was prepared by reacting 7 mM ABTS radical cation with 2.45 mM potassium persulphate at room temperature in the dark for 12–16 h. The solution was then diluted with distilled water to obtain an absorbance of 0.7 ± 0.02 at 734 nm. Aliquots (2 mL) of extracts at various concentrations (0.16–5 μg/mL) were then mixed with 2 mL of diluted ABTS radical cation solution. Absorbance was measured at 734 nm after incubating the samples at room temperature for 3 min. Ascorbic acid was used as a reference. ABTS radical cation scavenging activity was calculated using the same formula as that used to measure DPPH radical-scavenging activity. IC_50_ was determined by probit regression in SPSS (IBM, Armonk, NY, USA).

#### 4.5.3. Reducing Power Assay

The reducing power of the extracts was determined according to a previously described procedure [57] with some modifications. Extracts (1 mL) were mixed at various concentrations (0.78–50 μg/mL) with 2 mL of phosphate buffer (0.2 M, pH 6.6) and 1 mL of 1% potassium ferric cyanide [K_3_Fe(CN)_6_]. After incubation at 50 °C for 30 min, 2 mL of 10% trichloroacetic acid (TCA) was added. A portion of the solution (2 mL) was mixed with 2 mL of distilled water and 1 mL of 0.1% ferric chloride (FeCl_3_). The absorbance was then measured at 700 nm. Ascorbic acid was used as a reference. Ascorbic acid equivalent values were calculated from linear equations for the samples and reducing power was expressed as ascorbic acid equivalents (AAE) given in μg AAE/100 μg of the extracts.

#### 4.5.4. Assay for Metal Ion Chelating Activity

Fe^2+^-chelating activity was measured according to a previously described method [31] with some modifications. Extracts (1 mL) at various concentrations (0.31–10 mg/mL) were mixed with 0.2 mL of FeSO_4_ (1 mM) and 2.3 mL of distilled water. The mixture was shaken vigorously and left at room temperature for 5 min. Afterwards, 1 mL of ferrozine (1 mM in methanol) was added to the mixture, which was mixed and left for another 5 min to react with the residual Fe^2+^. The absorbance of the Fe^2+^ ferrozine complex was measured at 562 nm against a blank; EDTA-Na_2_ was used as a reference. Fe^2+^ chelating activity was calculated using the same formula as that used for the DPPH assay. IC_50_ was determined by probit regression in SPSS (IBM, Armonk, NY, USA).

### 4.6. In Vivo Antioxidant Activity Testing

#### 4.6.1. Animals

Specific-pathogen-free (SPF) male mice (20 ± 2 g) were purchased from the Experimental Animal Company of Chendu Dashuo (Chendu, China). Mice were allowed to acclimate to room conditions for three days prior to experimentation and were kept in a controlled environment at 25 ± 2 °C under a 12 h light/12 h dark cycle and in 60 ± 2% relative humidity. Mice were fed a standard rodent pellet diet and had access to fresh water at all times.

#### 4.6.2. Establishment of the Mouse Model of d-Galactose-Induced Aging

Mice were randomly divided into six groups, each containing ten mice. Group I was a blank control group that was subcutaneously injected with normal saline (100 mg/kg) once a day for six weeks. Groups II–VI were intraperitoneally injected with 100 mg/kg doses of d-galactose (0.1 mL/10 g) once a day for 6 weeks [33]. Group II was intragastrically administered with normal saline (100 mg/kg), and Group III was given ascorbic acid (100 mg/kg in saline water). Groups IV–VI were intragastrically administered with high (400 mg/kg), moderate (200 mg/kg), and low (100 mg/kg) doses of MF. Each group received intragastric administration for three weeks. Two days after the end of the experiment, the animals were anaesthetized through inhalation of ethyl ether. Eyeball blood was collected and allowed to clot, and the serum was separated for the assessment of enzyme activity. The mice were then sacrificed by cervical dislocation. Liver and brain samples were dissected, cleaned of blood with ice-cold saline, and immediately stored in a refrigerator for the determination of biochemical indices.

#### 4.6.3. Determination of Biochemical Indices

The collected eyeball blood was allowed to naturally sediment for 2 h. The resulting suspension was centrifuged at 3000 rpm for 10 min at 4 °C, and the supernatant was collected for 8-iso PGF_2α_ analysis. Liver and brain homogenates (10% *w*/*v*) were prepared in cold saline. The resulting suspension was centrifuged at 3000 rpm for 10 min at 4 °C and frozen at −80 °C until the assay was conducted as described below. The activities of 8-iso PGF_2α_ from blood serum, along with SOD, protein carbonyl, MDA, and GSH from liver and brain, were assayed using commercial reagent kits obtained from the Institute of Biological Engineering of Chengdu Kelong (Chengdu, China) according to the manufacturer’s instructions and using standard assay procedures [34,52].

### 4.7. DPPH-UHPLC-ESI-HRMSn Analysis

MF and ML extracts (5.0 mg/mL in methanol) were reacted with DPPH (0.2 mM in methanol) at 37 °C for 30 min; the volume ratio of the extracts and DPPH solutions was 1:1. The resulting mixtures were filtered through a 0.22 μm filter prior to UHPLC-ESI-HRMS^n^ analysis. A sample (2.5 mg/mL) without DPPH was used as a blank control. Peaks representing antioxidant components reduced or disappeared after reacting with DPPH. UHPLC analyses were performed using an Ultimate 3000 system (Dionex, Sunnyvale, CA, USA) equipped with an online vacuum degasser, a quaternary pump, an autosampler, and a temperature-controlled column compartment. Chromatographic separation was performed on a SunFireTM C_18_ (100 mm × 2.1 mm, 1.7 μm, Waters, MA, USA) column. The eluent consisted of solvents A (0.1% aqueous formic acid in water) and B (acetonitrile); flow rate was 0.3 mL/min. Gradient elution was programmed as follows: 0–2 min, 5% B; 2–10 min, 5%–15% B; 10–30 min, 15%–40% B; and 30–40 min, 40%–80% B. The injection volume was 2 μL, and the injection temperature was 15 °C.

Tandem mass spectrometry was performed in a Q-Exactive Orbitrap mass spectrometer (MS) (Thermo Fisher, Waltham, MA, USA) using a heated electrospray ionization source for the ionization of target compounds. MS data were acquired across a range of 80–1200 *m*/*z* in both positive and negative ion modes. The operating conditions were as follows: capillary voltage, 2.00 kV; pressure of nebulizer, 30 psi; auxiliary gas pressure, 10 L/min; capillary temp, 320 °C; auxiliary gas heater temp, 350 °C. MS/MS spectra were acquired with collision energy at 35 eV to achieve the maximum number of characteristic fragments for structural elucidation.

### 4.8. Statistical Analysis

All antioxidant experiments were performed in triplicate. SPSS (IBM, New York, NY, USA).and Graph Pad Prism were used to analyze the data statistically. Data were expressed as mean ± standard deviation (SD). The statistical evaluations used one-way analysis of variance (ANOVA) with multiple comparisons, followed by Dunnett’s *t*-tests, and the means compared using Duncan’s multiple range test. Statistical significance was determined at *p* < 0.05.

## 5. Conclusions

This study first evaluated the antioxidant activities of leaves and flowers from *Paeonia rockii* and revealed the presence of antioxidant compounds. The flowers and leaves from *Paeonia rockii* showed stronger antioxidant activity than ascorbic acid in vitro in reducing power, DPPH, and ABTS radical scavenging assay. The antioxidant ability of *Paeonia rockii* flowers was evidenced by the reduction of 8-iso PGF2α and protein carbonyl and a concurrent increase in SOD levels in vivo. In addition, 25 potential antioxidant compounds in the leaves and flowers were screened, and 18 compounds were identified by DPPH-UHPLC-ESI-HRMS^n^ methodology in vitro. The result demonstrated that the flavonoids and phenolic acids might be the major compounds responsible for the strong antioxidant activity of the flowers and leaves from *Paeonia rockii*. This study calls attention to the important antioxidant properties of *Paeonia rockii* and highlights the therapeutic potential of this traditional Chinese herb in preventing and treating diseases caused by oxidative stress.

## Figures and Tables

**Table 1:** Total phenolic content (TPC), total flavonoid content (TFC), and antioxidant activity of extracts prepared from *Paeonia* *rockii*.

Sample	Ascorbic Acid	EDTA-2Na	MF	ML
TPC (mg GAE/g extract)	-	-	540.38 ± 1.83	693.93 ± 0.82
TFC (mg RE/g extract)	-	-	130.40 ± 0.41	180.8 ± 0.37
DPPH (IC_50_, μg/mL)	3.21 ± 0.01	-	2.36 ± 0.00	1.50 ± 0.009
ABTS (IC_50_, μg/mL)	1.57 ± 0.21	-	1.03 ± 0.01	0.48 ± 0.01
Reducing power (μg AAE/100 μg extract)	100	-	119.85 ± 0.00	178.81 ± 0.00
Fe^2+^ chelation (IC_50_, mg/mL)	-	0.32 ± 0.00	7.95 ± 0.02	6.69 ± 0.00

GAE, gallic acid equivalents; RE, rutin equivalents; AAE, ascorbic acid equivalents. All values shown are the means of three determinations (mean ± SD); SD: standard deviation.

**Table 2:** Effects of *Paeonia rockii* flower extracts on 8-iso-PG from blood serum, brain and liver MDA, and protein carbonyl in d-galactose-induced mice.

Group	Dose/mg (mg/kg)	8-iso-PG/pg·mL^−1^	MDA/nmol·mg^−1^		Protein Carbonyl/nmol·mg^−1^	
			Brain	Liver	Brain	Liver
I	-	28.85 ± 5.10	9.82 ± 4.35	4.57 ± 3.59	49.25 ± 12.35	79.38 ± 16.52
II	-	46.49 ± 6.31 ^ΔΔ^	19.11 ± 4.46 ^ΔΔ^	5.37 ± 1.09 ^Δ^	118.51 ± 23.72 ^ΔΔ^	123.28 ± 12.12 ^ΔΔ^
III	100	30.74 ± 6.02 **	14.88 ± 5.83 *	4.5 ± 0.92 *	59.06 ± 22.36 **	88.94 ± 11.54 **
IV	400	33.35 ± 3.89 **	18.41 ± 5.62	4.23 ± 1.35	7.04 ± 0.93 *	15.35 ± 7.42 **
V	200	34.57 ± 3.37 **	10.69 ± 2.60 **	4.80 ± 1.92	6.58 ± 2.76 *	9.19 ± 6.68 **
VI	100	37.83 ± 2.03 *	14.01 ± 0.45 *	3.82 ± 1.67	9.65 ± 2.69 **	10.01 ± 4.44 **

Group I: Control, Group II: d-galactose-induced, Group III: Ascorbic acid 100 (mg/kg), Group IV: High 400 (mg/kg), Group V: Middle 200 (mg/kg), Group VI: Low 100 (mg/kg); ^Δ^ *p* < 0.05, ^ΔΔ^ *p* < 0.01 compared with the normal group; * *p* < 0.05, ** *p* < 0.01 compared with the d-galactose-induced group; All groups II–VI were d-galactose-induced. Data are given as the mean ± SD (*n* = 6).

**Table 3:** Effects of *Paeonia rockii* flower extracts on brain and liver SOD and GSH in d-galactose-induced mice.

Group	Dose/mg (mg/kg)	SOD/U·mg^−1^		GSH/nmol·mg^−1^	
		Brain	Liver	Brain	Liver
I	-	100.23 ± 32.57	96.85 ± 46.85	1.28 ± 0.26	0.73 ± 0.61
II	-	78.61 ± 26.09 ^ΔΔ^	64.31 ± 25.84 ^ΔΔ^	0.86 ± 0.14 ^Δ^	0.39 ± 0.27 ^ΔΔ^
III	100	100.91 ± 23.45 **	105.38 ± 41.66 **	1.41 ± 0.32 **	0.69 ± 0.22 *
IV	400	162.80 ± 30.12 *	89.10 ± 44.92 *	1.61 ± 1.27	0.44 ± 0.16
V	200	84.30 ± 27.44 *	98.54 ± 31.89 *	0.48 ± 0.08 **	0.35 ± 0.19
VI	100	104.85 ± 12.51 *	80.26 ± 33.37 **	0.67 ± 0.29 *	0.64 ± 0.72

Group I: Control, Group II: d-galactose-induced, Group III: Ascorbic acid 100 (mg/kg), Group IV: High 400 (mg/kg), Group V: Middle 200 (mg/kg), Group VI: Low 100 (mg/kg); ^Δ^ *p* < 0.05, ^ΔΔ^ *p* < 0.01 compared with the normal group; * *p* < 0.05, ** *p* < 0.01 compared with the d-galactose-induced group; All groups II–VI were d-galactose-induced. Data are given as the mean ± SD (*n* = 6).

**Table 4:** List of partially-identified antioxidant compounds from the flowers and leaves of *Paeonia rockii* by UHPLC-ESI-HRMS^n^ analysis.

Peak	T_R_ (min)	Formula	*m*/*z* Calculated	*m*/*z* Experimental	MS/MS Fragments	Proposed Compound	Flower	Leaf
1	1.47	C_7_H_6_O_5_	169.01315	169.01328	125, 97	Gallic acid	٭	٭
2	4.85	C_7_H_6_O_3_	137.02332	137.02327	93	Hydroxybenzoic acid	-	٭
3	5.65	C_8_H_8_O_5_	183.0288	183.02907	168, 124	Methyl gallate	٭	٭
4	6.44	C_20_H_20_O_14_	483.07693	483.07922	331, 313, 169, 125	Digalloyl glucose	٭	٭
5	12.57	C_34_H_28_O_22_	787.09885	787.10205	617, 465, 313, 169, 125	Tetragalloylglucose	٭	٭
6	12.93				336, 335, 183	Unknown	-	٭
7	13.15	C21H20O12	463.08710	463.08923	301, 257, 151	Quercetin-7-*O*-glucoside	٭	٭
8	13.15	C21H20O12	463.08710	463.08914	301, 300, 271, 255, 179, 151	Quercetin-3-*O*-glucoside	٭	٭
9	14.23	C_15_H_12_O_9_	335.03976	335.04150	183, 168, 124	Methyl digallate	-	٭
10	14.27	C28H24O15	599.10315	599.10571	447, 313, 285, 284, 169, 151, 125	Kaempferol galloylglucoside	-	٭
11	14.82	C_21_H_20_O_11_	447.09219	447.09396	285, 284, 255, 227, 179, 151	Astragalin	-	٭
12	15.27	C_21_H_20_O_11_	447.09219	447.09412	285, 284, 257, 151	Kaempferol-7-*O*-glucoside	-	٭
13	15.31	C22H22O12	477.10275	477.10468	357, 314, 285, 271, 257, 243, 151	Isorhamnetin-3-*O*-glucoside	-	٭
14	15.62	C_27_H_30_O_14_	577.15518	577.15741	431, 413, 269	Apigenin rhamnoglucoside	-	٭
15	15.78				469, 335, 190, 160, 146	Unkown	-	٭
16	16.35	C_28_H_24_O_14_	583.10823	583.11066	431, 313, 269, 169, 125	Apigenin galloylglucoside isomer	-	٭
17	19.10	C_28_H_24_O_14_	583.10823	583.11108	432, 431, 269, 268, 169, 125	Apigenin galloylglucoside isomer	٭	-
18	19.45	C_30_H_32_O_13_	599.17592	599.17865	477, 447, 431, 285, 281, 239, 179, 169, 149, 137, 121, 93	Benzoyloxypaeoniflorin	٭	٭
19	21.02				497, 461, 303, 160	Unkown	٭	-
20	21.48				483, 447, 187, 164, 160	Unkown	٭	-
21	21.92	C_15_H_10_O_5_	269.04445	269.04602	225, 159, 151, 117, 107	Apigenin	-	٭
22	23.18	C16H12O7	315.04993	315.18200	300, 271, 151	Isorhamnetin	٭	٭
23	23.95				190, 164, 160, 146, 132	Unkown	٭	-
24	24.25				269, 190, 187, 162, 160, 146, 132	Unkown	-	٭
25	38.26				143, 141, 136.134, 132, 121, 112	Unkown	-	٭

٭ There was a significant reduction after reaction with DPPH, - There was no significant reduction after reaction with DPPH.

## Abbreviations

TCM	traditional Chinese medicine
DPPH	2,2-diphenyl-1-picrylhydrazyl
ABTS	2,2′-azino-bis-(3-ethylbenzothiazoline-6-sulfonic) acid
UHPLC-ESI-HRMS^n^	ultra high pressure liquid chromatography and electrospray ionization coupled with high resolution mass spectrometry
8-iso-PGF2α	8-iso-prostaglandin F2α
SOD	superoxide dismutase
MDA	malondialdehyde
GSH	glutathione
ML	methanol extracts of *Paeonia rockii* leaves
MF	methanol extracts of *Paeonia rockii* flowers
TPC	total phenolic content
TFC	total flavonoid content
GAE	gallic acid equivalents
RE	rutin equivalents
TCA	trichloroacetic acid
AAE	ascorbic acid equivalents
EDTA-Na2	ethylenediaminetetraacetic acid disodium salt
tR	retention time
B-MF	Methanol extracts from flowers without reaction with DPPH
A-MF	Methanol extracts from flowers reacted with DPPH
B-ML	Methanol extracts from leaves without reaction with DPPH
A-ML	Methanol extracts of leaves after reaction with DPPH
SD	standard deviation
SPSS	Statistical Program for Social Sciences
